# Direct versus fully digital indirect bracket bonding: a split-mouth randomized clinical trial on accuracy

**DOI:** 10.1007/s00784-024-05950-6

**Published:** 2024-09-28

**Authors:** Pauline M.J. Hoekstra-van Hout, Jan Willem M. Hoekstra, Robin Bruggink, Ewald M. Bronkhorst, Edwin M. Ongkosuwito

**Affiliations:** 1 Department of Dentistry Section Orthodontics and Craniofacial Biology, Radboud university medical center, (THK 309), P.O. Box 9101, Nijmegen, 6500 HB The Netherlands; 2 Radboud University Medical Center, Radboudumc 3DLab, Nijmegen, The Netherlands

**Keywords:** Orthodontics, Bracket placement, Indirect bracket bonding, Computer-aided design, Chairside time, Fixed appliances

## Abstract

**Objectives:**

The primary aim is to assess differences in accuracy of orthodontic bracket positioning between fully digital indirect bracket bonding (IDB) and conventional direct bracket bonding (DBB). The secondary aims are to assess differences in bracket bonding failures, bracket repositioning need, clinician experience and patient satisfaction.

**Materials and methods:**

This prospective study was designed as a split-mouth randomized clinical trial. In total, 35 patients were analyzed with a six month follow-up period. Translational and orientational deviations from the planned bracket position were determined. Clinician experience and patient satisfaction were evaluated by means of a survey.

**Results:**

The difference in translation was 0.34 mm (95% CI: 0.238–0.352, *p* = 0.017), the difference in orientation was 4.80˚ (95% CI: 3.858–5.727, *p* < 0.001), both in favour of IDB. IDB showed significantly more immediate (IDB: 3.9%, DBB: 0%) and late (IDB: 5.4%, DBB: 2.5%, *p* = 0.008) bonding failures. Clinicians and patients experienced a shorter clinical chair time with indirect bonding over direct bonding.

**Conclusions:**

IDB bracket positioning leads to significant smaller translation and orientation deviations from digital IDB planning, than DBB bracket positioning. However, IDB leads to more immediate bonding failures than DBB. The majority of patients preferred IDB over DBB, due to a shorter clinical chair time.

**Clinical relevance:**

This study adds to the knowledge of IDB in orthodontics and contributes to evidence on this technique. This evidence is applicable in everyday orthodontics, with respect to patient satisfaction and technical limits of IDB. The trial was registered in the Dutch Trial Register and the International Clinical Trials Registry Platform (ICTRP) of the World Health Organization (WHO), number NL9411.

**Supplementary Information:**

The online version contains supplementary material available at 10.1007/s00784-024-05950-6.

## Introduction

In straight-wire orthodontics, precise and accurate bracket placement is of major importance for the clinical treatment outcome [1, 2]. In every-day orthodontic practice, brackets are placed with the direct bracket bonding (DBB) technique in most of the cases. Here, the correct bracket position is determined by the clinician during the bonding procedure, based on the clinical situation and treatment goals [2]. Positioning errors with this method are quite frequent, due to e.g. a limited view, anatomical difficulties or saliva contamination, which may result in a prolonged treatment time or a suboptimal treatment result [3, 4].

In an attempt to overcome the shortcomings of the DBB technique, an indirect bracket bonding (IDB) system was suggested for the first time by Silverman et al. in 1972 [5]. With this method, the brackets are placed on a plaster cast model. The advantage of extra-oral placement is a better view on the brackets and teeth, which makes it easier to place brackets in the correct position. The brackets are then transferred to the dentition of the patient with a transfer tray. This process with several technical steps is quite time-consuming [6]. With the emergence of computer-aided design and manufacturing (CAD/CAM), the number of intermediate steps can be reduced. Ciuffolo et al. presented a digital technique for IDB in 2006, in which a traditional impression is digitalized [7]. The three-dimensional (3D) digital model is part of a CAD/CAM process which is completely digital up to and including printing of the transfer tray, also referred to as ‘jig’ [7]. With current software and intra-oral scanning techniques, the last analogue step in IDB (i.e. impression taking) can also be digitalized. As such, Xue et al. described a fully digital procedure for IDB in 2020, using an intra-oral scanner, digital bracket planning and CAD/CAM transfer tray printing [8]. In this study by Xue et al. a high accuracy in bracket placement was found with deviations in mesiodistal, buccolingual, vertical, rotational and angulational bracket positioning well within the clinical acceptable range of 0.5 mm and 2.0° [8]. This study by Xue et al., a cohort study of 10 patients, however did not compare IDB to DBB [8].

A direct comparison between IDB and DBB with respect to bracket positioning accuracy and patient satisfaction is clinically significant. Incorrect bracket positioning in modern-day straight wire orthodontics with pre-adjusted appliances leads to prolonged treatment time, due to the need for either several wire bending steps or bracket rebonding [3, 4]. Further, incorrect bracket positioning with subsequent wire bending can lead to unwanted torque effects. As such, Meyer and Nelson calculated that a 3 mm vertical deviation of a bracket on a mandibular first premolar, leads to 15° torque discrepancy [9]. These torque issues are relatively difficult to resolve and can therefore lead to suboptimal treatment outcomes. Therefore any novel technical improvement that may lead to more precise bracket bonding, is of clinical importance.

IDB is associated with a significantly shorter clinical chair time compared to DBB [10]. However, the total time needed per patient (i.e. including digital bracket planning and transfer tray design) in IDB is longer compared to DBB [10]. Nonetheless, patients and clinicians may be more satisfied with IDB over DBB since less clinical chair time is needed.

To the best of our knowledge, no studies exist that report on a split-mouth randomized clinical trial comparing a fully digital IDB workflow with a conventional DBB workflow, including parameters such as clinician experience and patient satisfaction. The primary aim of this split-mouth randomized clinical trial is to assess differences in accuracy of bracket positioning of a fully digital IDB workflow compared to a DBB workflow, with digital IDB bracket planning serving as reference for both methods. The secondary aims are to assess differences between DBB and fully digital IDB, with respect to bracket bonding failures (immediate and delayed), bracket repositioning need (due to malpositioning), clinician experience and patient satisfaction.

## Materials and methods

### Study design

This prospective study was conducted in full accordance with regulations of the World Medical Association Declaration of Helsinki, and ICH E6 (R2) guidelines for Good Clinical Practice (GCP). Ethical approval of the local Human Research Ethics Committee was obtained (METC Oost-Nederland, number 2018–4032). The trial was registered in the Dutch Trial Register and the International Clinical Trials Registry Platform (ICTRP) of the World Health Organization (WHO), number NL9411. The manuscript was prepared according to CONSORT guidelines [11].

The study was designed as a split-mouth randomized clinical trial, with a randomized block design with a 1:1 allocation of IDB and DBB. The randomization list was generated by a biostatistician and patients were allocated based on order of inclusion.

In every participating patient the first arch which was planned to be bonded (i.e. upper or lower jaw), was included in the study. The decision on which arch had to be bonded first in a particular patient, was based on clinical features and the patient’s individual treatment plan. Allocation of the left and right side to either IDB or DBB was based on randomization. The other arch was also orthodontically treated with fixed appliances but not included in the study. The clinical part of the study could not be blinded due to obvious differences in bracket placement procedures between IDB and DBB. The follow-up period was six months after bonding.

### Sample size calculation

The standard deviation (SD) of the of the maximum linear positioning error is 0.037 [12]. For uncorrelated errors this would give a standard deviation of 0.052 for the difference in position errors in one pair of brackets. As these errors are most likely correlated, the SD will be lower. How strong that reduction is, depends on the size of the correlation, which we do not know. We assume the SD for the difference in positioning errors in one pair of brackets to be 0.045. Aiming for a power of 0.80 to detect a difference in positioning errors of 0.01, with an alpha of 0.05 we need to include 159 pairs of brackets in our study. With on average six pairs of brackets per patient, that would imply 27 patients. However, since six pairs of brackets are placed in each patient we have to correct for the loss of power due to a cluster of multiple pairs within one patient. The sample size was therefore corrected by a factor 1 + interclass correlation coefficient (ICC) * (cluster size − 1). The cluster size is 6, the ICC is unknown so we assume it to be about 0.05. That asks for an increase of the sample size by 25%, resulting in a final number of 34 patients to be included in this study. To account for a ~ 10% drop-out, the final sample size was set at 37.

### Patient selection

All patients referred to the department of Dentistry, section of Orthodontics and Craniofacial Biology of the Radboud university medical center between January 2021 and December 2021, were screened for the following in- and exclusion criteria.

#### Inclusion criteria

- Healthy patient (ASA score I).
- Permanent dentition present from at least first molar to first molar.
- Vestibular fixed appliances indicated in both arches.
- Bonding indicated at least from second premolar to second premolar.

#### Exclusion criteria

- Agenesis (congenitally missing teeth).
- Extractions indicated.
- (Pre)molar bands indicated.
- Syndromes or enamel abnormalities present.

All patients received verbal and written information about the study. Written informed consent was obtained for participating in this study from all patients and/or their legal guardians in case of minors.

### Bracket planning and transfer tray design

**Fig. 1:**
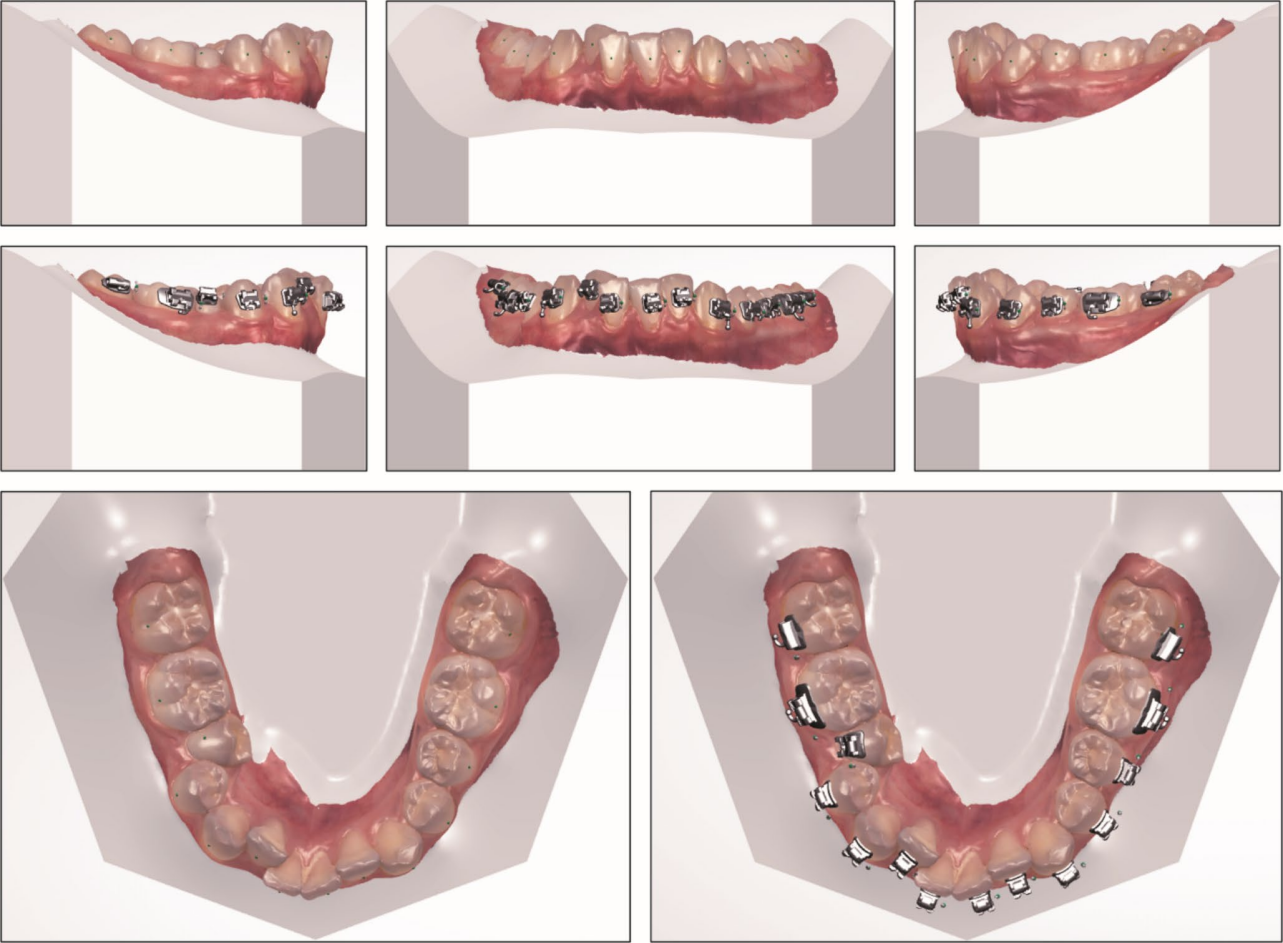
Example of digital indirect bracket planning, the green dots on non-bonded teeth represent the facial axis points of the teeth

Intra-oral scans of all patients were obtained with a TRIOS 3 intra-oral scanner (3Shape A/S, Copenhagen, Denmark). Scans were imported in 3Shape Ortho Analyzer software (v2021). On these digital models, preadjusted self-ligating brackets (In-Ovation R, Dentsply Sirona, NYC, USA) with a bidimensional slot size (.018” from canine to canine and 0.022” for (pre)molars) were digitally placed on all teeth to be bonded (see Fig. 1), i.e. both on the IDB and on the DBB side. All brackets were initially digitally positioned by the software and then manually adjusted to an ideal position. Digital bracket planning was performed for all patients by one clinician (author PH) and then checked and if necessary corrected by one of four clinical faculty members (licensed orthodontists) before the transfer trays were designed and printed.

The transfer trays were designed in 3Shape Appliance Designer software (v2020). The applied settings for the design were:

- 0.0 mm offset.
- 0.0 degrees block-out angle.
- 0.1 mm retention amount.
- 1.0 mm prolongation length.

**Fig. 2:**
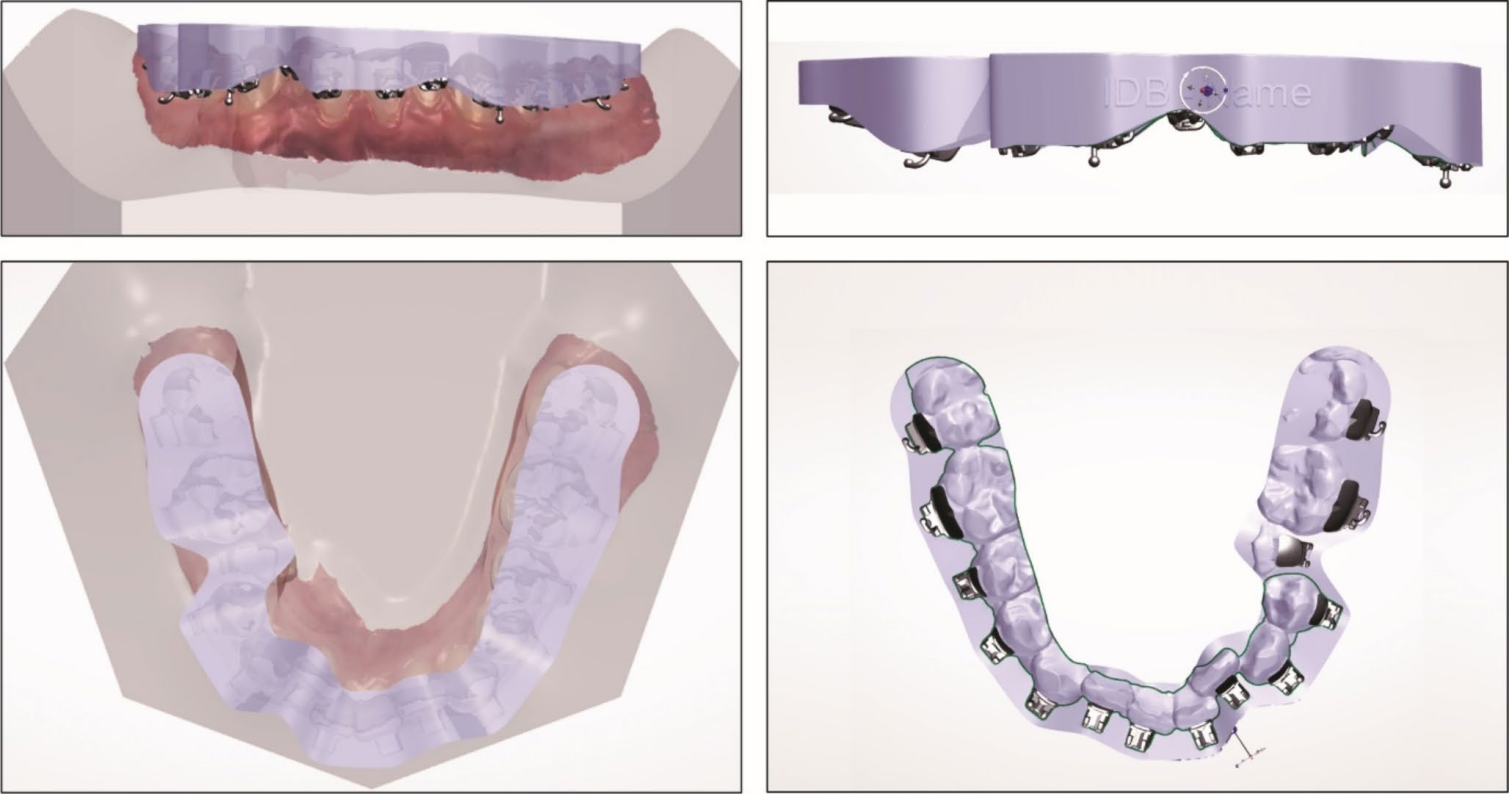
Example of digital transfer tray design

All transfer trays were designed to provide full occlusal support (see Fig. 2). The bracket housing was designed both on the IDB as well as on the DBB side. The brackets were fully covered by the transfer tray, except for the 0.5 mm most cervical aspects of the brackets. The transfer trays were printed by an external orthodontic appliances laboratory (Cordent B.V., Maartensdijk, the Netherlands), with an Asiga Max UV printer (Asiga, Alexandria, NSW, Australia), with a 150 μm printing resolution, using NextDent Ortho IBT biocompatible class I transparent printing resin (NextDent B.V., Soesterberg, the Netherlands) with a shore A hardness of 85.

### Bracket bonding

All transfer trays for all patients were prepared shortly before treatment by the same clinician who performed digital bracket planning and transfer tray design (PH). First the transfer trays were cut in half so that only the IDB side of the tray was used, the other half was disposed of. Brackets were then placed in the transfer tray. All brackets were prepared with resin composite (Transbond XT, 3 M, St. Paul, MN, USA) by one clinician (PH) to avoid intra-clinician differences in resin composite thickness. The transfer trays with brackets were then stored in a dark drawer until treatment of the patient commenced (maximum storage time from resin composite application to treatment was three hours).

Bonding (both IDB and DBB) was performed by a total of seven postgraduate residents in Orthodontics at the Radboud university medical center, Nijmegen, the Netherlands. The clinical bonding procedure was supervised by the same faculty member who supervised the digital bracket planning for that particular patient. In all patients, IDB was performed first and DBB was performed second to avoid vertical linear differences in the incisor area.

After obtaining a dry field by isolation (Nola Dry Field System, Greatlakes Ortho, Tonawanda, NY, USA), all teeth were buccally etched with 37% phosphoric acid (DMG, Hamburg, Germany) and primed with Transbond XT adhesive (3 M, St. Paul, MN, USA). The bracket transfer tray was then transferred to the mouth of the patient, firmly pressed into place and checked for correct placement. Excess resin composite was removed at the accessible cervical aspect of the brackets, succeeded by light-curing of the resin composite. A light-emitting diode polymerization lamp with a minimal output of 1.000 mW/cm^2^ was used and the bracket transfer tray was then removed. Thereafter DBB was performed on the contralateral side. All DBB brackets were placed one by one with Transbond XT resin composite applied by the clinician directly before bracket placement, excess resin composite was removed, brackets were checked for the correct position and finally the resin composite was light-cured. All patients received either a round 0.012” or 0.014” NiTi initial archwire depending on the clinical need/situation. After the bonding procedure, an intra-oral scan was made for positioning evaluation purposes. Further, patients received oral hygiene instructions and specific fixed appliance instructions, as standard of care.

### Pilot study

Due to possible great technique sensitivity in design and preparation of the transfer trays, a pilot study was performed on six patients. In this way, several different transfer tray designs and the amount of resin composite were tested before the correct configuration was determined and then used in the main study.

### Technical study parameters

The technical study parameters were as follows:

- Accuracy of bracket placement.
- Immediate and delayed bonding failures.
- Bracket repositioning need.

**Fig. 3:**
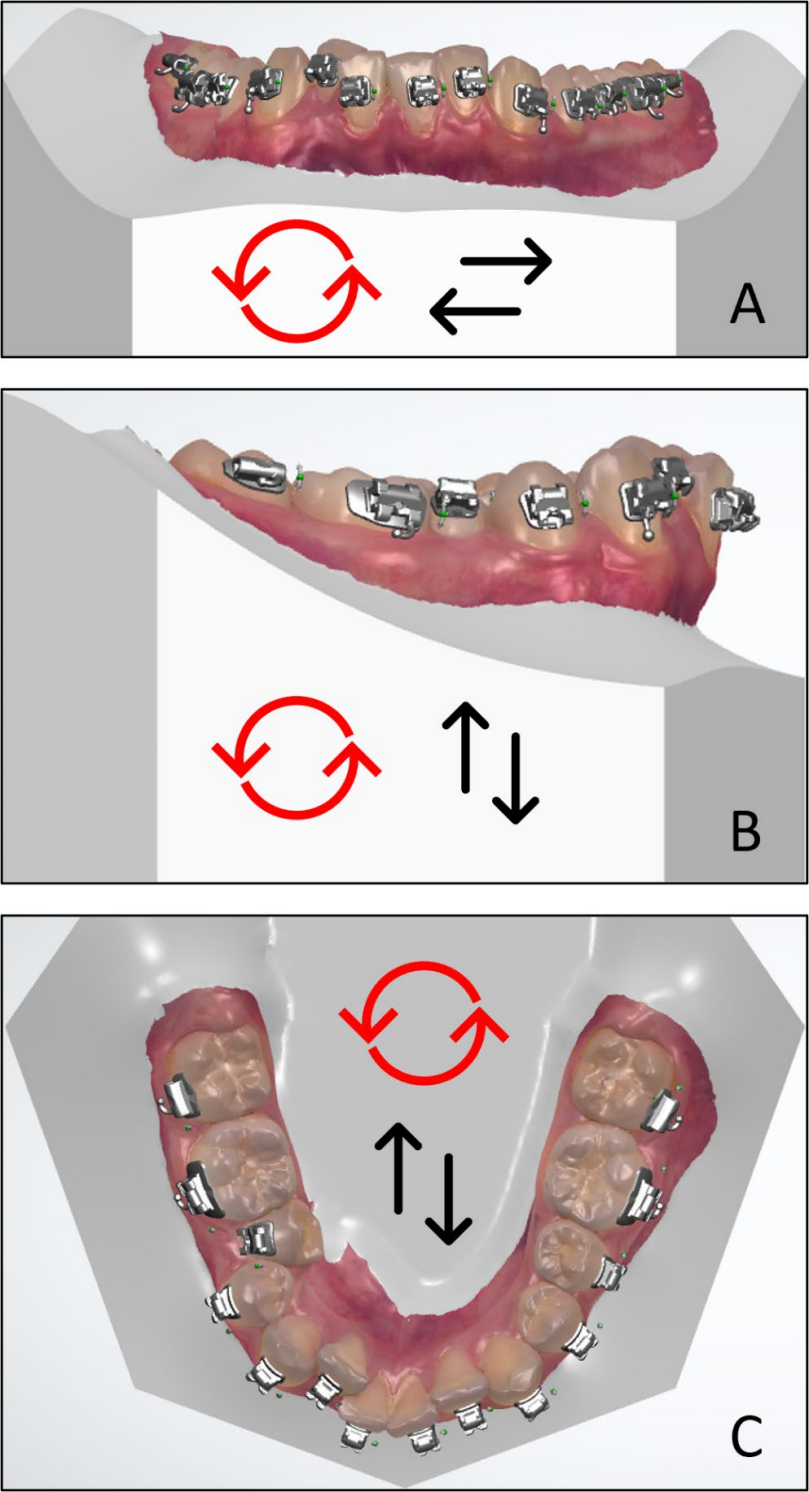
**A** = Roll and X-axis; **B** = Pitch and Y-axis; **C** = Yaw and Z-axis. Roll, pitch and yaw in red arrows; X, Y and Z in black arrows

The accuracy of clinical bracket placement compared to digital IDB bracket planning for both IDB and DBB, was determined by comparing the 3D files of the immediately post-bonding intra-oral scans with the 3D digital bracket planning files. For this purpose, the scanning and design files were exported as Standard Tessellation Language (STL) files and imported into in-house-created software, 3DMedX^®^, which is based on Open Inventor software (version 9.9.19, Houston, TX, USA). The accuracy measurement consisted out of three steps. At first, the post-bonding and the digital planned model where initially aligned with respect to their palate using an iterative closest point based algorithm (ICP). Secondly, the regions of the teeth, excluding the brackets, were manually selected and used to align each individual tooth of the planned model towards the post-bonding model. At this moment the differences between the planned and performed placement of the brackets were visualized. Finally, the bracket locations were automatically determined and used to align each tooth with respect to the bracket. After visual inspection, the linear differences (X, Y, Z axes) and rotational differences (roll, pitch, yaw [R, P, Y]) of the last alignment were automatically determined by the software (see Fig. 3). In the remainder of this article, ‘linear’ and ‘rotational’ will be referred to as ‘translational’ and ‘orientational’, respectively. For analytical reasons the three deviations for translation (X, Y and Z) were added together, as well as the three deviations for orientation (R, P and Y), giving two accuracy outcomes for each bracket placed. In order to correctly sum the X/Y/Z and R/P/Y values, Euclidean values were used (i.e. negative values were converted into positive values).

All immediate IDB and DBB bonding failures were scored at the bonding appointment. For a time period of six months after bonding, delayed bracket bonding failures and the need for bracket repositioning due to positioning errors were scored.

### Intra- and inter-observer agreement

All measurements in 3DMedX^®^ were performed by one author (PH). Precision of measurements was evaluated by re-measurements of the first 10 patients by PH (intra-observer agreement). Trueness of the measurements by PH was assessed by means of an inter-observer agreement test, where the first 10 patients were also measured by another author (RB) and compared to the measurements of PH.

To ensure the measurements, authors PH and RB made agreements on beforehand about which regions of the teeth were to be manually selected to align each individual tooth of the planned model towards the post-bonding model. All other steps in the measurement process were fully automatically performed by the software, hence no agreements needed to be made by PH and RB about these steps.

The intra- and inter-observer agreement values were expressed in three ways, i.e. as the correlation, as the structural difference with a 95% confidence interval and *p*-value and as the duplicate measurement error (DME).

### Clinician experience and patient satisfaction survey

Both clinician experience and patient satisfaction were evaluated by a short qualitative questionnaire designed for this study (see Online Resources 1 and 2). All seven postgraduate students who participated in this study were queried about their experiences with IDB after completion of the clinical phase of the study. All participating patients were queried about their satisfaction before and immediately after bonding.

Questions about the clinician experience covered transfer tray fitting, resin composite issues (amount, excess, curing), bracket positioning, clinical treatment time, learning curve and general experience.

In the patient satisfaction survey prior to treatment, patients were asked if they were looking forward to receiving fixed appliances and getting straight teeth. Items evaluated immediately after bonding were clinical treatment time (both IDB and DBB), preference for either IDB or DBB and general remarks.

### Quality of life assessment

To characterize all patients, Oral Health Related Quality of Life (OHRQoL) was evaluated pre-treatment at inclusion in the study when obtaining informed consent. The questionnaire used for evaluation of OHRQoL was the Dutch version of the FACE-Q Dental module (Q Portfolio, Canada). The items evaluated were categorized into nine domains divided over three scales; appearance appraisal scales (domains: general [10 questions], face [15 questions], smile [16 questions], teeth [20 questions] and jaws [14 questions]), oral function scales (domain: eating and drinking [13 questions]), and quality of life scales (domains: school life [10 questions, only for patients 8–18 years of age], social life [11 questions] and psychological wellbeing [10 questions]). Each question was scored on a four-point scale and all scores were added together to form a total score per domain. The total domain scores were thereafter converted into equivalent Rasch scores ranging from 0 to 100 (respectively low and high quality of life).

### Statistical analysis

For quantitative data analysis, SPSS software (version 27, IBM, Armonk, NY, USA) and R (version 4.1.3, R Foundation, Vienna, Austria) were used.

For each pair of brackets, on the same type of tooth though left-right mirrored, e.g. on tooth 35 and tooth 45, this study gives a pair of accuracies: one for a DBB bonded bracket and one for an IDB bonded bracket (both compared to digital IDB planning). The differences between these accuracies within each pair (always DBB minus IDB) was the unit of analysis. As primary analysis, the accuracy (for both translation as well as orientation) was compared using a multilevel regression model with a random intercept for patient and no independent variables. This is equivalent to a paired t-test, but with the extension that it corrects for the fact that there are multiple pairs in one patient. As secondary analysis, arch and type of tooth (incisor, canine, premolar, molar) were added as independent variables to the regression model.

Qualitative analysis was performed for the clinician experience and patient satisfaction surveys. Further, absolute values and percentages were calculated for immediate and delayed bracket bonding failures, and bracket repositioning need due to positioning errors. The immediate and delayed bonding failures and bracket repositioning need were further evaluated by means of a McNemar chi-square test for paired data.

## Results

### Patients and treatments

Initially 37 patients were included between January 2021 and December 2021, of which two dropped out of the study. One drop-out was caused by a partly erupted canine that could not be bonded and the other drop-out was caused by a non-fitting transfer tray. Hence, a total of 35 patients were analyzed in the study, of which 15 (43%) were male and 20 (57%) female. The mean age of the patients was 14 years (SD 5 years), with a range of 9 to 33 years. In total, 203 IDB and 203 DBB brackets were used for analysis. Left and right division and upper and lower arch division of IDB and DBB, were almost equal (see Table 1). Further, the amount of brackets and distribution over the patients is presented in Table 2. OHRQoL FACE-Q scores at pre-treatment are presented in Table 3.

Apart from the previously mentioned non-fitting transfer tray, no adverse events occurred during treatment. In general, IDB brackets appeared to present more resin composite residue after light-curing compared to DBB brackets, albeit still clinically acceptable.

**Table 1:** Number of patients treated and distribution of IDB / DBB left and right

	Upper arch	Lower arch	Total
**IDB L / DBB R**	9	10	19
**IDB R / DBB L**	7	9	16
**Total**	16	19	35

**Table 2:** Distribution of brackets

	Upper arch	Lower arch	Total
**Bonding 7–7**	2	9	11
**Bonding 6–6**	4	8	12
**Bonding 5–5**	10	2	12
**Total**	16	19	35

**Table 3:** Rasch scores per FACE-Q domain, with 0 as minimum and 100 as maximum with respect to quality of life

		Mean		SD	Range
**Appearance appraisal scales**					
General		77.08		13.03	46–100
Face		58.31		10.84	37–84
Smile		56.83		14.13	23–100
Teeth		54.23		14.80	0–91
Jaws		63.03		22.34	0–100
**Oral function scales**					
Eating and drinking		84.39		13.22	42–100
**Quality of life scales**					
School life (8–18 yrs)		72.11		13.19	50–100
Social life		73.46		14.54	48–100
Psychological wellbeing		74.94		19.33	32–100

### Accuracy of bracket bonding

The intra-observer agreement measurements showed a correlation of 0.98 and 0.96 for translation and orientation values, respectively. The structural difference for translation values was 0.00 mm (95% CI [-0.02–0.01], *p* = 0.65) and for orientation values 0.05 mm (95% CI [-0.23–0.33], *p* = 0.73). The DME was 0.07 mm for translation and 1.13° for orientation values.

For the inter-observer agreement, the correlation for translation and orientation values was 0.92 and 0.82, respectively. The structural difference for translation values was − 0.01 mm (95% CI [-0.04–0.03], *p* = 0.71) and for orientation values − 0.41 mm (95% CI [-0.99–0.18], *p* = 0.17). The DME was 0.14 mm for translation and 2.36° for orientation values.

The accuracy of bracket placement is shown in Table 4. The summed X/Y/Z and R/P/Y values were used for statistical analysis. DBB showed a higher deviation in clinical bracket position compared to the digitally planned bracket positions, than IDB brackets did.

The analyses of the difference in accuracy of bracket placement between IDB and DBB are shown in Table 5. The primary analysis showed a statistically significantly better accuracy for IDB both for translation as well as for orientation. The difference in translation was 0.34 mm in favour of IDB (95% CI: 0.24–0.35, *p* = 0.017). In orientation, the difference was 4.80˚ (95% CI: 3.86–5.73, *p* < 0.001) in favour of IDB.

When taking into account the dental arch itself and the position in the arch, as secondary analysis, the difference between IDB and DBB was not statistically significantly influenced by the arch. However, there was clearly an effect of place in the dental arch. For both translation and orientation, the difference was smallest with incisors. There too, the difference between IDB and DBB was statistically significant in favour of IDB. Further distally, the difference between IDB and DBB statistically significantly increased.

**Table 4:** Accuracy of bracket placement

	Mean	SD	Min	Max
**Translation accuracy (mm)**				
IDB X	0.09	0.09	0.00	0.56
DBB X	0.21	0.18	0.00	0.85
IDB Y	0.18	0.18	0.00	0.91
DBB Y	0.27	0.23	0.00	1.09
IDB Z	0.11	0.11	0.00	0.72
DBB Z	0.25	0.26	0.00	1.32
IDB (sum of X + Y + Z)	0.38	0.27	0.03	1.74
DBB (sum of X + Y + Z)	0.73	0.46	0.09	2.69
**Orientation accuracy (degrees)**				
IDB R	0.87	0.90	0.00	7.58
DBB R	3.13	2.79	0.00	14.33
IDB P	1.19	1.01	0.00	5.33
DBB P	2.68	2.54	0.01	18.64
IDB Y	1.35	1.32	0.01	7.13
DBB Y	2.43	2.18	0.03	11.10
IDB (sum of R + *P* + Y)	3.40	2.15	0.27	13.13
DBB (sum of R + *P* + Y)	8.24	5.15	0.97	34.20

**Table 5:** Primary and secondary analysis of difference in accuracy between DBB and IDB. All differences are calculated as DBB minus IDB. Results from multilevel regression analysis

**Primary analysis**			
	DBB – IDB	95% CI	*p*
Translation accuracy (mm)	0.34	0.238–0.436	< 0.001
Orientation accuracy (degrees)	4.80	3.86–5.73	< 0.001
**Secondary analysis**			
*Translation (mm)*	DBB – IDB	95% CI	*p*
Reference (lower incisor)	0.20	0.04–0.35	0.017
Upper arch (vs. lower)	-0.06	-0.26–0.14	0.545
Canine (vs. incisor)	0.20	0.03–0.38	0.024
Premolar (vs. incisor)	0.21	0.07–0.35	0.004
Molar (vs. incisor)	0.41	0.23–0.59	< 0.001
*Orientation (degrees)*	DBB – IDB	95% CI	*p*
Reference (lower incisor)	2.32	0.75–3.89	0.005
Upper arch (vs. lower)	-0.24	-2.10–1.61	0.799
Canine (vs. incisor)	3.07	1.06–5.08	0.003
Premolar (vs. incisor)	4.29	2.65–5.93	< 0.001
Molar (vs. incisor)	3.96	1.89–6.06	< 0.001

### Bonding failures and repositioning need

For IDB brackets, immediate bracket bonding failure occurred in three patients (8.6%) for a total of eight brackets (3.9%). Of these eight debonded brackets, four occurred in one patient for which the clinician noted a high lip-pressure and difficulty of maintaining dry field isolation. For the DBB brackets no immediate bracket bonding failures occurred, hence a McNemar chi-square test could not be performed.

Late IDB bracket bonding failures occurred in nine patients (25.7%) for a total of 11 brackets (5.4%). Late DBB bracket bonding failures occurred in four patients (11.4%) for a total of five brackets (2.5%). The McNemar chi-square test at bracket-level revealed a significant difference between IDB and DBB (*p* = 0.008).

Repositioning of IDB brackets was needed in three patients (8.6%) for a total of five brackets (2.5%). For DBB brackets, repositioning was needed in two patients (5.7%) for a total of two brackets (1.0%). No significant difference between IDB and DBB was present in the McNemar chi-square test at bracket-level (*p* = 0.125).

### Clinician experience and patient satisfaction survey

The total clinician experience survey, including all answers given, is presented as Online Resource 1. In general, clinicians experienced a shorter clinical chair time with indirect bonding over direct bonding (four clinicians ‘agreed’ on this statement, three ‘strongly agreed’). Transfer trays were often fitting correctly (six clinicians mentioned ‘often’, one mentioned ‘always’), light curing was almost always uneventful (six mentioned ‘always’ and one mentioned ‘sometimes’) and clinicians were often satisfied with the eventual bracket position (five mentioned ‘often’, one mentioned ‘sometimes’ and one mentioned ‘always’). There were however some issues with abundance of excess composite which could not be removed with the transfer tray in place (three mentioned ‘always’ and four mentioned ‘often’). All clinicians agreed (four clinicians) or strongly agreed (three clinicians) that a learning curve exists for using the indirect bonding technique. In total, four clinicians had no preference for either IDB or DBB, two clinicians preferred DBB and one clinician preferred IDB. Nevertheless, upon asking if the clinicians would probably use IDB in the future, five clinicians answered ‘yes’, one answered ‘no’ and one answered ‘maybe’. Frequently mentioned prerequisites for using IDB in the future were that the digital planning phase does not take a large amount of time of the orthodontist and that the costs for the transfer trays are not too high.

The total patient satisfaction survey, including all answers given, is presented as Online Resource 2. Almost all patients were looking forward to having straight teeth and had neutral attitude towards the placement of the fixed appliance. Total treatment time and total bonding time (IDB and DBB together) was experienced as ‘medium’ by the vast majority of patients (25 and 28 out of 35, respectively). However, the treatment time for IDB was experienced as ‘short’ (24 out of 35) and the treatment time for DBB was scored as ‘medium’ by the majority of patients (19 out of 35). Two patients noticed no difference between the two treatment techniques. Of the remaining 33 patients, 25 patients preferred IDB over the DBB. The most frequently mentioned reason for preferring IDB, was the reduced amount of treatment time (scored 20 times). The second most mentioned reason was that IDB was experienced as a more easy method (scored 6 times). The eight patients who preferred DBB, most frequently mentioned that the transfer tray had a very tight fit, leading to pain and discomfort (scored three times) during removal of the tray.

## Discussion

This prospective split-mouth randomized clinical trial aimed to assess differences in accuracy of bracket positioning of a fully digital IDB workflow compared to a DBB workflow. Secondly, differences between IDB and DBB with respect to bracket bonding failures, bracket repositioning need, clinician experience and patient satisfaction were assessed. Brackets placed by the DBB technique showed significantly more deviation from the digital IDB planning than brackets placed by the IDB technique. On the other hand, the IDB workflow showed more immediate and late bracket bonding failures than the DBB workflow. Bracket repositioning need was almost equal for IDB and DBB. Clinicians and patients experienced a shorter clinical chair time with indirect bonding compared to direct bonding, leading to a preference for IDB amongst the vast majority of patients. Abundance of excess composite which could not be removed with the transfer tray in place, was the most frequently mentioned issue by clinicians with the IDB protocol.

One of the first studies on IDB was published by Silverman et al. in 1972 [5]. With the emergence of CAD/CAM, Ciuffolo et al. [7] presented a digital technique for IDB in 2006 (albeit with traditional impression taking and subsequent digitalization of models) to overcome the time-consuming technical laboratory steps of conventional IDB [6]. With the development of 3D intra-oral scanning and digital workflows, a fully digital procedure for IDB is now possible. As such, Xue et al. [8] and Palone et al. [12] performed prospective cohortstudies of respectively 10 and 17 patients with a fully digital workflow including intra-oral scanning, though without comparison to the conventional DBB technique. To the best of our knowledge, our study is the first to report a randomized clinical trial comparing a fully digital IDB workflow to a conventional DBB workflow, including parameters such as clinician experience and patient satisfaction.

The results of IDB bracket placement accuracy in our study showed a summed translation accuracy of 0.38 ± 0.27 mm for IDB and 0.73 ± 0.46 mm for DBB. The mean orientation accuracy was 3.40 ± 2.15° for IDB and 8.24 ± 5.15° for DBB. Compared to the previous mentioned studies by Xue et al. [8] (up to 0.09 ± 0.05 mm and 0.29 ± 1.28°) and Palone et al. [12] (up to 0.02 ± 0.02 mm and 0.46 ± 0.54°), our angular deviations seem to be relatively large. However, our values are expressed as the sum of individual X/Y/Z and R/P/Y values, whereas the values in Xue et al. [8] and Palone et al. [12] are expressed as individual X/Y/Z and R/P/Y values. We added these values together for statistical reasons, giving two accuracy outcomes for each bracket placed. Averaging X/Y/Z and R/P/Y values or working with maximum individual deviations instead of with summed deviations, would imply weakening of data and statistical analysis. Still, our mean individual X/Y/Z values lie well within the clinical acceptable range of 0.5 mm translation [8]. Our mean individual R/P/Y values however do exceed the clinical acceptable range of 2° orientation [8], though only for DBB brackets. Further, in vitro studies and IDB studies with traditional impression taking show angular deviations up to 3.36° [13, 14].

Analysis of the difference between IDB and DBB bracket placement with respect to positioning, showed a significantly more precise positioning of IDB brackets compared to DBB brackets for both translation and orientation values. Although significant, the differences were not clinically relevant for translation values (0.34 mm). On the other hand, the orientation difference between IDB and DBB of 4.80˚ is quite large. This is however a summed value of mean individual R/P/Y values. When subtracting mean individual R, P and Y IDB values from DBB values, the difference does not exceed 2.26° which is less though still clinically important. This indicates that judging a correct angulation of the bracket is more difficult with DBB than with IDB, especially further distally in the mouth as confirmed by the multilevel regression analysis in our study.

Accuracy of DBB bracket placement was measured in this study with the IDB bracket planning as an ideal bracket positioning model. To have the IDB planning serve as a golden standard for DBB placement, was also performed by Koo et al. [14]. In our study the ideal digital IDB bracket placement was corrected by one of four clinical faculty members with each a different view on what an ideal bracket position is, mainly in occlusal-apical direction. To overcome the risk of incorporating differences between digital IDB planning and clinical DBB placement due to personal preferences, the clinical bonding procedure was supervised by the same faculty member who supervised the digital bracket planning for that particular patient. The results show that the mean translation deviation in the vertical Z-axis of DBB bonding compared to IDB planning was 0.25 ± 0.26 mm, indicating that faculty members have little difficulty distinguishing between a digital and clinical situation.

Ideal IDB bracket planning was performed on digital models with unresolved malocclusions. This means that achieving the ideal digital bracket positions was possibly hampered by crowding. An alternative method to overcome this problem, is digital treatment of the malocclusion with subsequent digital placement of brackets in the ideal position, and finally reverting this planning to the original malocclusion with the digital brackets in place. This method was applied by several studies [13–16], however not in our study since this does not represent the clinical case. Assessing differences between digital and clinical bracket positioning, as investigated in our study, would be impossible with this method. Further, this method has limitations in cases where severe crowding hampers adequate bracket positioning.

Immediate bracket bonding failure occurred in our study in 3.9% of IDB brackets and 0% of DBB brackets. These values are comparable to those reported in previous studies [10, 17, 18]. Late bonding failures occurred in 5.4% of IDB brackets and 2.5% of DBB brackets (*p* = 0.008) in our study. Other studies reported similar bonding failures rates for DBB [20], and IDB [17], although IDB and DBB showed no differences in late bonding failures between the two techniques in those and other studies [17–20]. A possible explanation for the difference in immediate and late bonding failures between IDB and DBB is a less effective light curing through the transfer trays, which is known to have an effect on bracket shear bond strengths [21]. Indeed, Thiyagarajah et al. found no difference in immediate bonding failures between IDB and DBB with the use of a very thin completely transparent Essix transfer tray, ensuring effective light-curing [19]. Immediate bonding failure leads to a prolonged chair time and a new bracket can only be placed through direct bracket bonding, since replacement of the transfer tray is hard or even impossible after removal. The potential benefit of IDB is then lost for those teeth. Late bonding failure potentially prolongs total treatment time, depending on the phase of treatment, which leads to decreased patient satisfaction and reduces cost-effectiveness. Transfer tray design is therefore pivotal for the potential success of IDB and needs to be optimized in future research.

The clinical chair time of IDB was experienced as shorter compared to DBB chair time, by both clinicians and patients. This is confirmed by the results of Czolgosz et al. [10] and a systematic review and meta-analysis by Li et al. [21]. However, the total time needed for bonding, (i.e. including digital planning and tray design) is longer in IDB compared to DBB [10, 21], with the digital bracket planning phase taking 54% of the total IDB time [10]. The shorter clinical chair time was probably the reason for patients to prefer IDB over DBB (25 out of 35 patients), nevertheless only one clinician out of seven preferred IDB over DBB. In our study the executing clinicians did not perform the time-consuming digital bracket planning, we feel therefore that unfamiliarity with the new IDB procedure was most likely the reason that only one clinician preferred IDB over DBB. Another reason could be that IDB showed a significant higher immediate bonding failure rate. The higher rate of immediate bonding failures in IDB increases the total treatment time of IDB and reduces the clinical practicality.

Two limitations of our study need to be mentioned, potentially biassing the results. First, this study could obviously not be blinded for clinicians and patients. We have tried to minimize the risk of investigator bias, by almost fully automizing bracket positioning analysis by the software. Immediate and late bracket debonding are dichotomous parameters, for which the risk of investigator bias is limited. The judgement for bracket rebonding need due to malpositioning is most prone to investigator bias, although no significant differences between IDB and DBB were found for that parameter. Secondly, the fact that IDB was always performed before DBB could potentially have biased the results by introducing a sequencing effect. We chose to adhere to this order, based on results of the pilot study. Here we saw a step in vertical bracket positioning between the two central incisors when DBB was performed before IDB. All in all, we feel that the (potential) bias in our study was fairly low.

Future research should focus on cost-effectiveness of indirect bonding versus direct bonding. Although the total treatment time of IDB is longer than the total treatment time of DBB, this does not mean that the net time for the orthodontist is longer per sé. In a setting where dental assistants are allowed to bond brackets, the orthodontist has to control and correct DBB bonding procedures before light curing to ensure correct bracket positioning. In case of IDB, this chair time by the orthodontist could possibly be replaced by digital bracket positioning time. The orthodontist then no longer has to check the clinical bonding procedure, possibly leading to more efficient clinical time. With recent developments in artificial intelligence, the digital bracket positioning procedure could possibly further be optimized leading to a decrease of time investment in the digital phase of IDB.

## Conclusions

This prospective split-mouth randomized clinical trial showed that IDB bracket positioning leads to significant smaller translation and orientation deviations from digital IDB planning, than DBB bracket positioning. Mainly the difference between IDB and DBB with respect to orientation of brackets is clinically relevant. Further, IDB leads to more immediate bonding failures than DBB. Last, IDB showed shorter clinical chair time compared to DBB, which led to a preference of the majority of patients for IDB over DBB.

## Electronic supplementary material

Below is the link to the electronic supplementary material.

Supplementary Material 1

Supplementary Material 2

## Data Availability

Data is provided within the manuscript and supplementary information files. All data underlying this article will be shared on reasonable request to the corresponding author.

## References

[1] Andrews LF (1972) The six keys to normal occlusion. Am J Orthod 62:296–309. 10.1016/s0002-9416(72)90268-0 4505873 10.1016/s0002-9416(72)90268-0

[2] McLaughlin RP, Bennet JC (1995) Bracket placement with the preadjusted appliance. J Clin Orthod 29:302–311 PMID: 8617853. No DOI available 8617853

[3] Carlson SK, Johnson E (2001) Bracket positioning and resets: five steps to align crowns and roots consistently. Am J Orthod Dentofac Orthop 119:76–80. 10.1067/mod.2001.111220 10.1067/mod.2001.111220 11174544

[4] Miethke RR, Melsen B (1999) Effect of variation in tooth morphology and bracket position on first and third order correction with preadjusted appliances. Am J Orthod Dentofac Orthop 116:329–335. 10.1016/s0889-5406(99)70246-5 10.1016/s0889-5406(99)70246-5 10474107

[5] Silverman E, Cohen M, Gianelly AA, Dietz VS (1972) A universal direct bonding system for both metal and plastic brackets. Am J Orthod 62:3. 10.1016/s0002-9416(72)90264-3 10.1016/s0002-9416(72)90264-3 4559001

[6] Layman B (2019) Digital Bracket Placement for Indirect Bonding. J Clin Orthod 53:387–396 PMID: 31648214. No DOI available 31648214

[7] Ciuffolo F, Epifania E, Duranti G, De Luca V, Raviglia D, Rezza S et al (2006) Rapid prototyping: a new method of preparing trays for indirect bonding. Am J Orthod Dentofac Orthop 129:75–77. 10.1016/j.ajodo.2005.10.005 10.1016/j.ajodo.2005.10.005 16443482

[8] Xue C, Xu H, Guo Y, Xu L, Dhami Y, Wang H et al (2020) Accurate bracket placement using a computer-aided design and computer-aided manufacturing-guided bonding device: an in vivo study. Am J Orthod Dentofac Orthop 157:269–277. 10.1016/j.ajodo.2019.03.022 10.1016/j.ajodo.2019.03.022 32005479

[9] Meyer M, Nelson G (1978) Preadjusted edgewise appliances: theory and practice. Am J Orthod 73:485–498. 10.1016/0002-9416(78)90239-7 354404 10.1016/0002-9416(78)90239-7

[10] Czolgosz I, Cattaneo PM, Cornelis MA (2021) Computer-aided indirect bonding versus traditional direct bonding of orthodontic brackets: bonding time, immediate bonding failures, and cost-minimization. A randomized controlled trial. Eur J Orthod 43:144–151. 10.1093/ejo/cjaa045 32780096 10.1093/ejo/cjaa045

[11] Schulz KF, Altman DG, Moher D (2010) CONSORT Group. CONSORT 2010 statement: updated guidelines for reporting parallel group randomised trials. BMJ 340:c332. 10.1136/bmj.c332 20332509 10.1136/bmj.c332 PMC2844940

[12] Palone M, Koch PJ, Jost-Brinkmann PG, Spedicato GA, Verducci A, Pieralli P et al (2022) Accuracy of indirect bracket placement with medium-soft, transparent, broad-coverage transfer trays fabricated using computer-aided design and manufacturing: an in-vivo study. Am J Orthod Dentofac Orthop 12:S0889–5406. 10.1016/j.ajodo.2021.08.023 10.1016/j.ajodo.2021.08.023 36243597

[13] Kim J, Chun YS, Kim M (2018) Accuracy of bracket positions with a CAD/CAM indirect bonding system in posterior teeth with different cusp heights. Am J Orthod Dentofac Orthop 153:298–307. 10.1016/j.ajodo.2017.06.017 10.1016/j.ajodo.2017.06.017 29407508

[14] Koo BC, Chung CH, Vanarsdall RL (1999) Comparison of the accuracy of bracket placement between direct and indirect bonding techniques. Am J Orthod Dentofac Orthop 116:346–351. 10.1016/s0889-5406(99)70248-9 10.1016/s0889-5406(99)70248-9 10474109

[15] Park JH, Choi JY, Oh SH, Kim SH (2021) Three-Dimensional Digital Superimposition of Orthodontic Bracket position by using a computer-aided transfer Jig System: an Accuracy Analysis. Sens (Basel) 21:5911. 10.3390/s21175911 10.3390/s21175911 PMC8434098 34502801

[16] Oliveira NS, Gribel BF, Neves LS, Lages EMB, Macari S, Pretti H (2019) Comparison of the accuracy of virtual and direct bonding of orthodontic accessories. Dent Press J Orthod 24:46–53. 10.1590/2177-6709.24.4.046-053.oarli 10.1590/2177-6709.24.4.046-053.oar PMC6733233 31508706

[17] Aguirre MJ, King GJ, Waldron JM (1982) Assessment of bracket placement and bond strength when comparing direct bonding to indirect bonding techniques. Am J Orthod 82:269–276. 10.1016/0002-9416(82)90461-4 6760721 10.1016/0002-9416(82)90461-4

[18] Bozelli JV, Bigliazzi R, Barbosa HA, Ortolani CL, Bertoz FA, Faltin Junior K (2013) Comparative study on direct and indirect bracket bonding techniques regarding time length and bracket detachment. Dent Press J Orthod 18:51–57. 10.1590/s2176-94512013000600009 10.1590/s2176-94512013000600009 24351150

[19] Thiyagarajah S, Spary DJ, Rock WP (2006) A clinical comparison of bracket bond failures in association with direct and indirect bonding. J Orthod 33:198–204. 10.1179/146531205225021615 16926313 10.1179/146531205225021615

[20] Li Y, Mei L, Wei J, Yan X, Zhang X, Zheng W et al (2019) Effectiveness, efficiency and adverse effects of using direct or indirect bonding technique in orthodontic patients: a systematic review and meta-analysis. BMC Oral Health 19:137–148. 10.1186/s12903-019-0831-4 31286897 10.1186/s12903-019-0831-4 PMC6615229

[21] Rachala MR, Yelampalli MR (2010) Comparison of shear bond strength of orthodontic brackets bonded with light emitting diode (LED). Int J Orthod 21:31–35. PMID 21314086. No DOI available. 21314086

