# Supplementary material for: Direct versus fully digital indirect bracket bonding: a split-mouth randomized clinical trial on accuracy
Source: Clin Oral Investig. 2024 Sep 28;28(10):557. doi: 10.1007/s00784-024-05950-6 (PMC11438723; doi:10.1007/s00784-024-05950-6)
Supplement: Supplementary file 1 — Supplementary Material 1 [file 784_2024_5950_MOESM1_ESM.docx]

Direct versus fully digital indirect bracket bonding: a split-mouth randomized clinical trial on accuracy. Clinical Oral Investigations. Pauline M.J. Hoekstra-van Hout; Jan Willem M. Hoekstra; Robin Bruggink; Ewald M. Bronkhorst; Edwin M. Ongkosuwito. Radboud University Medical Center, Department of Dentistry, section Orthodontics and Craniofacial Biology, Nijmegen, the Netherlands. [Janwillem.hoekstra@radboudumc.nl](mailto:Janwillem.hoekstra@radboudumc.nl)

Indirect Bonding: Clinician Experience Survey

| **What is your opinion on chair time?** | **Very short** | **Short** | **Medium** | **Long** | **Very long** |
| --- | --- | --- | --- | --- | --- |
| 1) Total chair time *One side direct bracket bonding, other side indirect bracket bonding* |  | 2 | 5 |  |  |
| 2) Only indirect bracket bonding chair time | 1 | 5 | 1 |  |  |

|  |  | | | | |
| --- | --- | --- | --- | --- | --- |
| **INDIRECT BONDING** | | | | | |
|  | | **Never** | **Sometimes** | **Often** | **Always** |
| 3) The transfer trays were fitting accurately | |  |  | 6 | 1 |
| 4) There was a sufficient amount of composite on the bracket pads | |  |  | 3 | 4 |
| 5) There was an abundance of excess composite around the brackets after curing | |  | 3 | 4 |  |
| 6) Curing of composite was uneventful | |  | 1 |  | 6 |
| 7) I was satisfied with the position of the brackets | |  | 1 | 5 | 1 |

| **INDIRECT VS. DIRECT BONDING** | | | | | |
| --- | --- | --- | --- | --- | --- |
|  | **Strongly disagree** | **Disagree** | **Neutral** | **Agree** | **Strongly agree** |
| 8) Indirect bracket bonding took less time than direct bracket bonding |  |  |  | 4 | 3 |
| 9) I think I will be faster in indirect bracket bonding if I treat more patients |  |  | 3 | 2 | 2 |
| 10) Indirect bracket bonding is more pleasant than direct bracket bonding |  | 2 | 4 | 1 |  |
| 11) The transfer tray was easy to use |  | 1 | 3 | 3 |  |
| 12) I quickly became proficient in using the transfer tray |  |  |  | 6 | 1 |
| 13I think I would have become more skilled in using the transfer tray if I had bonded more patients using the indirect bonding method |  |  |  | 4 | 3 |

14) a. Which method did you prefer? Circle your preference.

A. Direct bonding method: 2

B. Indirect bonding method: 1

C. No preference: 4

14) b. Please explain your answer to the previous question.

- *A1: The direct method is more certain. Bracket position is adapted to the tooth form in direct bonding, this is not the case in indirect bonding. As a result, there is more composite present between bracket and tooth surface in the indirect bracket bonding method. There is more excess composite around the brackets with the indirect method. Less composite use leads to bonding failures. Indirect bonding is faster in chair time, but digital planning is a huge time investment. In case of an immediate bonding failure, the bracket needs to be rebonded in a direct bonding way, this takes time. All in all, direct bonding is probably faster. Some trays were not fitting accurately. I would prefer to design the trays in sextants instead of quadrants.*
- *A2: Even though both methods work, I slightly prefer direct bonding for two reasons: 1) the excess composite and 2) the inability to place brackets slightly under the gingiva in case of partly erupted teeth.*
- *B1: The clinical procedure of indirect bonding was pleasant for me. Correct placement of brackets is more predictable and takes less time compared to direct placement. Movement of the patient is not a problem in indirect bonding.*
- *C1: Both methods work fine. Indirect bonding only adds something if the entire arch is bonded indirectly.*
- *C2: Sometimes indirect bonding works fine, sometimes it doesn’t. Sometimes the fitting of the tray is not adequate and excess resin composite can not be removed. But if everything works fine and there are no technical issues, the indirect method is definitely fast and easy. However if there are technical issues with the indirect method, the direct bonding is absolutely faster.*
- *C3: Both methods have pro’s and con’s. Chair time is faster with indirect, but the digital planning takes a lot of time. I think indirect bonding is useful in patients with a limited cooperation. Delegation of bracket placement to assistants is easier with the indirect method and saves time for the orthodontist. There is a learning curve in clinical use.*
- *C4: First time indirect bonding I experienced three immediate debonds. That was not a good start. Second time was better, but I was struggling with correct placement of the transfer tray. Third time everything went well. So I have no real preference, although I think that indirect placement is the future.*

15) a. Do you want to use the indirect bonding method in the future? Circle your preference.

Yes 5

No 1

Maybe 1

15) b. Please explain your answer to the previous question.

- *Yes 1: Only if it is already in the workflow of the orthodontic practice. Check of bonding by an assistant is not necessary anymore. One should apply the technique regularly to become and stay skillful in this method.*
- *Yes 2: In case of patients with limited cooperation I think I would prefer the indirect bonding method.*
- *Yes 3: If I get more experienced in the indirect method, I think this is faster than the direct bonding technique. The only disadvantages are the learning curve and the digital planning time. But on the long run, assistants can do bondings independent, without the orthodontist, since the planning is performed by the orthodontist digitally on beforehand.*
- *Yes 4: Only if all technical problems are solved and the method is more predictable. But the technical issues do not mean that the indirect method is not adequate. If printing techniques improve and if excess composite can adequately be removed, this is a promising method.*
- *Yes 5: I would use indirect bonding if the planning phase is easy and if additional costs are not too high. The increased comfort and reduced chair time of indirect bonding are big advantages for both the patient and the orthodontist. However, the time savings for the practitioner should not be negated by the time required to make the planning.*
- *No 1: I see no indication for the use of indirect bracket bonding. If assistants bond brackets and the orthodontist checks the positioning before curing the composite, there is no great time saving for the orthodontist with the indirect bonding method. Further, planning / computer time investment is huge and costs are higher due to the transfer tray.*
- *Maybe 1: Including planning, indirect is not faster than direct bonding, but costs are higher and storage of trays takes space. The trays incorporate an extra factor in the treatment, with possibly an extra error. If I would use indirect bonding, I would bond both arches at the same time, to minimize time between the intra-oral scan and the tray production/usage.*

16) Do you have any additional remarks?

*No answers*
