# Supplementary material for: Direct versus fully digital indirect bracket bonding: a split-mouth randomized clinical trial on accuracy
Source: Clin Oral Investig. 2024 Sep 28;28(10):557. doi: 10.1007/s00784-024-05950-6 (PMC11438723; doi:10.1007/s00784-024-05950-6)
Supplement: Supplementary file 2 — Supplementary Material 2 [file 784_2024_5950_MOESM2_ESM.docx]

Direct versus fully digital indirect bracket bonding: a split-mouth randomized clinical trial on accuracy. Clinical Oral Investigations. Pauline M.J. Hoekstra-van Hout; Jan Willem M. Hoekstra; Robin Bruggink; Ewald M. Bronkhorst; Edwin M. Ongkosuwito. Radboud University Medical Center, Department of Dentistry, section Orthodontics and Craniofacial Biology, Nijmegen, the Netherlands. [Janwillem.hoekstra@radboudumc.nl](mailto:Janwillem.hoekstra@radboudumc.nl)

Indirect Bonding: Patient Satisfaction Survey

**Research: Placing fixed braces with or without a fitting tray**

Dear participant,

Today you will get your fixed braces. Before the braces are placed, we kindly ask you to fill out the first page of this survey. Directly after the treatment, the clinician will ask you to fill out the second page. Thank you for participating in this study!

**BEFORE PLACEMENT OF THE FIXED BRACES
Answer each question by putting a checking one box**

|  | **Not at all** | **No** | **Neutral** | **Yes** | **Very much** |
| --- | --- | --- | --- | --- | --- |
| 1. Are you looking forward to having straight teeth? |  |  | **2** | **15** | **18** |
| 2. Are you looking forward to the period with your fixed braces? | **2** | **6** | **23** | **2** | **2** |
| 3. Are you looking forward to today’s placement of your braces? |  | **2** | **21** | **12** |  |

**AFTER PLACEMENT OF THE FIXED BRACES**

**Answer each question by checking one box.**

| ***What do you think of the time needed for…*** | **Very short** | **Short** | **Medium** | **Long** | **Very long** |
| --- | --- | --- | --- | --- | --- |
| 1. …the total treatment today? |  | **8** | **25** | **2** |  |
| 1. …bonding all brackets (left and right) |  | **5** | **28** | **2** |  |
| 1. …bonding brackets without the tray |  | **6** | **19** | **9** | **1** |
| 1. …bonding brackets with the tray | **4** | **24** | **7** |  |  |

|  | **Yes** | **No** |
| --- | --- | --- |
| 5. Did you notice any difference between bonding of the brackets (left and right)? | **33** (Proceed with question 6) | **2** (Proceed with question 8) |

|  | **Without tray** | **With tray** |
| --- | --- | --- |
| 6. Which treatment did you prefer? | **8** | **25** |

| 7. Why did you prefer that treatment? | *Answers: see next page* |
| --- | --- |

| 8. Do you have any additional comments about the treatment? | *Answers: see next page*  **2** |
| --- | --- |

**Answers to question 7:**

Participants prefering indirect bonding:

- All brackets placed at once (1x)
- Faster (20x)
- Easier (6x)
- Less pressure on teeth (1x)

Participants prefering direct bonding:

- Removing the tray was painful (3x)
- The tray was very tight and painful to the gums (1x)
- The tray was tight and painful to the teeth (1x)
- The tray was tight
- Removal of the tray was difficult (1x)

**Answers to question 8:**

- Curing was more difficult with the tray (1x)
- All went well (1x)
- Removal of the tray was painful (1x)
- Removal of the tray was uncomfortable (1x)
